# Effect of bisphosphonates on orthodontic tooth movement: an umbrella review

**DOI:** 10.1186/s12903-026-08984-2

**Published:** 2026-07-02

**Authors:** Serena Amin, Maria Cremona, Stefan Abela

**Affiliations:** 1 Department of Orthodontics, Norfolk and Norwich University Hospitals, Norwich, UK; 2 Private Practice, St. Paul’s Bay, Malta; 3 Department of Physics of Medicine, University of Cambridge, Cambridge, UK

**Keywords:** Bisphosphonates, Orthodontic tooth movement, Root resorption, Anchorage, Systematic review, Umbrella review

## Abstract

**Background:**

Bisphosphonates (BPs) are potent inhibitors of bone resorption widely used to manage metabolic bone disorders including osteoporosis, Paget’s disease, and malignant bone disease. With a growing number of adult patients undergoing orthodontic treatment while receiving BP therapy, understanding the interaction between these medications and orthodontic tooth movement (OTM) is of clinical importance.

**Objective:**

To synthesise the evidence from published systematic reviews on the effect of bisphosphonates on orthodontic tooth movement, orthodontic anchorage, and root resorption.

**Materials and methods:**

A systematic search of MEDLINE via OVID, PubMed, EMBASE, Scopus, Web of Science, the Cochrane Database of Systematic Reviews, PsycINFO, and AMED was conducted up to 31st March 2026. Systematic reviews examining the effect of BPs on OTM, root resorption, or anchorage were eligible for inclusion. Two reviewers independently performed selection, data extraction, and quality assessment using AMSTAR 2. Overlap across included reviews was assessed using a Corrected Covered Area (CCA) matrix. Within this umbrella review, animal and human evidence were synthesised separately.

**Results:**

Seven systematic reviews were included, comprising one review of human patients and six reviews of animal studies, published between 2010 and 2025. The overall CCA was approximately 6.1%, indicating slight to moderate overlap. Two reviews were rated critically low and five were rated moderate on AMSTAR 2. All included reviews reported reduced OTM following BP administration. Risedronate showed the most consistent inhibitory effect in animal studies. One review conducted a meta-analysis and found BPs to be significantly more effective than osteoprotegerin in reducing mesiodistal OTM in animals. The human-focused review reported slower tooth movement and compromised outcomes in extraction cases. Evidence on root resorption was contradictory across reviews.

**Conclusions:**

Available evidence, derived predominantly from animal studies, consistently indicates that BPs reduce OTM. Human evidence remains very limited and is insufficient to support firm clinical recommendations. Well-designed prospective human studies are needed to clarify the clinical implications of BP use during orthodontic treatment.

**Trial registration:**

PROSPERO registration: CRD42023372111

**Supplementary Information:**

The online version contains supplementary material available at https://doi.org/10.1186/s12903-026-08984-2.

## Introduction

Bisphosphonates (BPs) are a class of synthetic pyrophosphate analogues that act as potent inhibitors of bone resorption. They are most commonly used to treat metabolic bone diseases including osteoporosis, Paget’s disease, hyperparathyroidism, and malignancies involving skeletal metastases [1]. Their primary mechanism of action involves inhibition of osteoclastic activity either through promotion of osteoclast apoptosis or by reducing the ability of osteoclasts to adhere to bone surfaces undergoing active resorption [2]. On resorption of the surrounding bone matrix, the stored BP is released and prevents the osteoclast from generating the protons required for further breakdown [3, 4].

Orthodontic tooth movement (OTM) is dependent on bone remodelling in response to applied mechanical forces [5]. Since BPs directly modulate osteoclastic activity, they have a clear biological mechanism through which they may interfere with the bone remodelling cycle and alter the rate or extent of tooth movement [6]. This has raised interest from two clinical perspectives: first, whether systemic BP use constitutes a risk factor for compromised orthodontic outcomes in patients receiving these medications; and second, whether targeted local BP application might be used therapeutically to enhance anchorage control or limit unwanted tooth movement during orthodontic treatment [7, 8].

Previous individual systematic reviews have explored these questions but have generally been constrained by heterogeneous study designs, reliance on animal data, and small sample sizes [9, 10]. Several systematic reviews now address overlapping aspects of this question, yet they vary in methodological quality and reach partially conflicting conclusions, particularly regarding root resorption. This makes a higher-level synthesis appropriate to appraise and integrate the existing reviews systematically. This paper therefore presents an umbrella review of available systematic review evidence, analysing animal and human findings separately to allow appropriate interpretation at each level of evidence. The primary outcome was to summarise evidence on the effect of BPs on OTM; secondary outcomes were the effect on orthodontic anchorage and root resorption.

## Materials and methods

This review required no ethical approval as it involved no patient or animal participation and no personal data collection.

### Protocol and registration

The protocol was registered with PROSPERO: Registration number CRD42023372111. This umbrella review was reported in accordance with the Preferred Reporting Items for Systematic Reviews and Meta-Analyses (PRISMA) guidelines and the Preferred Reporting Items for Overviews of Reviews (PRIOR) statement.

### Eligibility criteria

The following PICO framework was applied:

- Population: Human patients or animal models with orthodontic appliances in situ.
- Intervention: Administration of bisphosphonate medications of any type, dose, or route, for any duration.
- Comparison: Control or placebo group, or subjects not receiving bisphosphonates.
- Primary outcome: Effect of BPs on OTM. Secondary outcomes: Effect on orthodontic anchorage and root resorption.
- Study design: Systematic reviews, with or without meta-analysis.

### Search strategy

A comprehensive electronic database search was conducted across PubMed, MEDLINE via OVID, EMBASE, the Cochrane Database of Systematic Reviews, AMED, PsycINFO, Scopus, and Web of Science. The search was updated to 31st March 2026. The strategy combined controlled vocabulary terms (MeSH and EMTREE subject headings) with free-text terms relating to bisphosphonates and OTM (Supplementary Table 1). A manual search of five orthodontic journals was also performed: the European Journal of Orthodontics, the American Journal of Orthodontics and Dentofacial Orthopaedics, the British Dental Journal, the Journal of Orthodontics, and The Angle Orthodontist. PROSPERO was searched for registered but unpublished reviews, and reference lists of included studies were hand-checked. No language or date restrictions were applied.

### Study selection

Two authors (SSA and SA) independently screened all titles and abstracts identified by the search. Full texts of potentially eligible studies were then assessed independently by the same two reviewers. Disagreements at both stages were resolved by discussion or referral to a third author (MC).

### Data extraction

Two authors (SSA and SA) independently extracted the following data from each included review: author, publication year, study design, country of origin of included primary studies, number of primary studies, sample size, type of primary studies, risk of bias assessment tool, databases searched, period of search, journal of publication, review objective, AMSTAR 2 rating, type and route of BP administered, method of measuring OTM, main findings, and limitations. Disagreements were resolved by MC. Full data extraction is provided in Supplementary Table 2.

### Overlap assessment

The degree of overlap of primary studies across included reviews was assessed using a Corrected Covered Area (CCA) matrix (Table 1). CCA values are interpreted as slight (< 5%), moderate (6–10%), high (11–15%), or very high (> 15%).

**Table 1:** Corrected Covered Area (CCA) matrix: overlap of primary studies across included systematic reviews

Review	Zymperdikas 2020	Iglesias-Linares 2010	Fernandez-Gonzalez 2015	Harikrishnan 2022	Makrygiannakis 2019	Miranda 2024	Sarango-Quishpe 2025
Zymperdikas 2020 [11]	--	0	0	0	0	0	0
Iglesias-Linares 2010 [12]	0	--	2	2	0	2	3
Fernandez-Gonzalez 2015 [13]	0	2	--	3	0	1	2
Harikrishnan 2022 [14]	0	2	3	--	0	2	3
Makrygiannakis 2019 [15]	0	0	0	0	--	0	0
Miranda 2024 [16]	0	2	1	2	0	--	2
Sarango-Quishpe 2025 [17]	0	3	2	3	0	2	--

Numbers represent the count of primary studies shared between each pair of reviews. Overall CCA was estimated at approximately 6.1%, indicating slight to moderate overlap, which is considered acceptable. Zymperdikas et al. [11]) included only human clinical studies and had no overlap with any animal-focused review. CCA thresholds: slight < 5%, moderate 6–10%, high 11–15%, very high > 15%

### Quality assessment

The methodological quality of all included reviews was assessed using AMSTAR 2, a validated 16-item tool [18]. Assessments were performed independently by SSA and SA, with disagreements resolved by MC. AMSTAR 2 ratings were integrated into the interpretation of findings throughout the results and discussion sections to contextualise the strength of conclusions.

### Data synthesis

Given the substantial heterogeneity across included reviews in terms of study design, BP type, dosage, route of administration, species studied, and outcome measures, meta-analysis at the level of this umbrella review was not feasible. Findings across the included reviews were therefore synthesised structurally. To maintain appropriate hierarchical interpretation, findings from animal and human studies are reported separately under each thematic heading. Themes were defined a priori based on the primary and secondary outcomes: (1) methodological quality and risk of bias; (2) methods of assessing tooth movement; (3) type of BP and method of administration; (4) sagittal and vertical tooth movement; (5) orthodontic anchorage; (6) root resorption [19, 20].

## Results

### Search and study selection

**Fig. 1:**
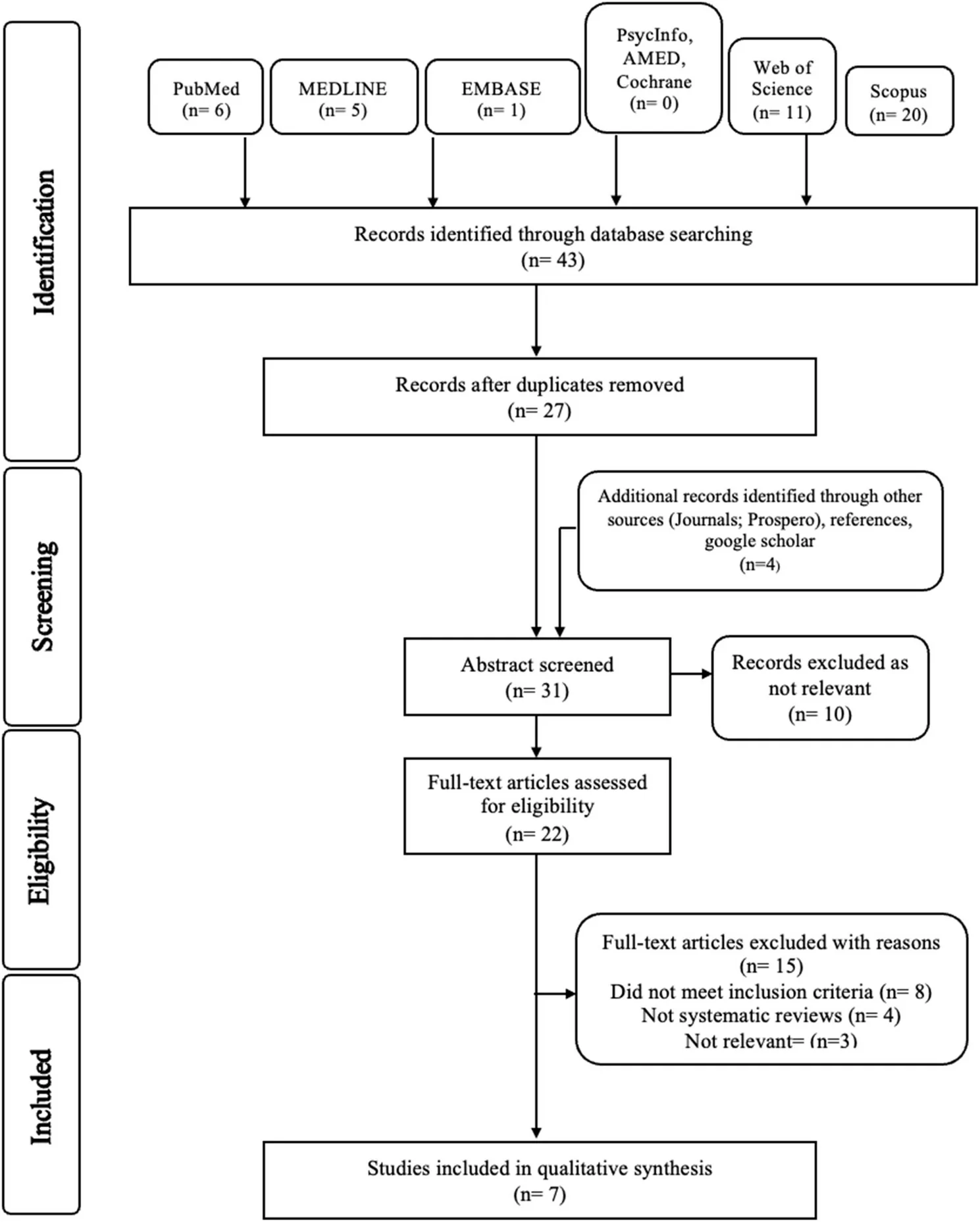
PRISMA flow diagram of literature selection process

The initial electronic database search identified 43 potentially eligible studies. Following removal of duplicates, 27 studies proceeded to title and abstract screening. Four additional studies were identified through supplementary sources including manual journal searching, PROSPERO, and Google Scholar. Twenty-two articles were assessed at full text. Fifteen were excluded: eight did not meet the inclusion criteria, four were not systematic reviews, and three were not relevant to the review question (Supplementary Table 3). This resulted in seven systematic reviews for inclusion ( Fig. 1 in Appendix).

### Study characteristics

The seven included systematic reviews were published between 2010 and 2025. Six were systematic reviews without meta-analysis; one also incorporated a meta-analytic component. Table 2 summarises the key characteristics of the included reviews. Full data extraction is provided in Supplementary Table 2.

**Table 2:** Summary of characteristics of included systematic reviews

Author	Year	Design	No. studies	Type of studies	Period of search	Journal	AMSTAR 2 rating
Zymperdikas et al.	2020	Systematic review	7	1 retrospective cohort study; 6 case reports (human patients)	Up to March 2019	Eur J Orthod	Moderate
Iglesias-Linares et al.	2010	Systematic review	13	13 experimental animal studies (rats)	Up to December 2008	J Dent	Critically low
Fernandez-Gonzalez et al.	2015	Systematic review	11	11 case-control animal studies (rats and mice)	2000 to July 2014	Dental Press J Orthod	Critically low
Harikrishnan and Ramasamy	2022	Systematic review	7	7 case-control animal studies (rats, rabbits, mice)	Up to May 2022	J Orthod Sci	Moderate
Makrygiannakis et al.	2019	Systematic review	21 (2 relevant to BPs)	Animal studies; 2 assessed bisphosphonates	Until April 2018	Eur J Orthod	Moderate
Miranda et al.	2024	Systematic review	3	3 experimental animal studies (rodents)	April 2020, updated June 2023	Dental Press J Orthod	Moderate
Sarango-Quishpe et al.	2025	Systematic review and meta-analysis	12 (SR); 7 (MA)	12 animal studies (healthy animals; rats and mice)	Up to August 2023	Korean J Orthod	Moderate

A distinction in study populations is fundamental to interpreting all findings that follow. Six of the seven included reviews [12–17] included exclusively animal studies, predominantly in rats and mice. Only one review [11] assessed human orthodontic patients.

### Overlap assessment

The CCA matrix (Table 1) showed an overall CCA of approximately 6.1%, indicating slight to moderate overlap. The most notable overlap was between the three reviews focused on local BP administration and anchorage [12–14], which shared two to three primary animal studies. The 2025 review by Sarango-Quishpe et al. [17] also shared three primary studies with both Iglesias-Linares et al. [12] and Harikrishnan and Ramasamy [14]. Zymperdikas et al. [11] had no overlap with any animal-focused review as it contained only human clinical studies. This level of overlap is considered acceptable.

### Methodological quality (AMSTAR 2)

AMSTAR 2 results are presented in Tables 3 and 4. Two reviews were rated critically low [12, 13] and five were rated moderate [11, 14–17]. No review achieved a high rating.

**Table 3:** AMSTAR 2 overall confidence rating categories

Rating	Criteria
High	No or one non-critical weakness: the systematic review provides an accurate and comprehensive summary of the results of the available studies that address the question of interest.
Moderate	More than one non-critical weakness: the systematic review has more than one weakness but no critical flaws. It may provide an accurate summary of the results of the available studies that were included in the review.
Low	One critical flaw with or without non-critical weaknesses: the review has a critical flaw and may not provide an accurate and comprehensive summary of the available studies that address the question of interest.
Critically low	More than one critical flaw with or without non-critical weaknesses: the review has more than one critical flaw and should not be relied on to provide an accurate and comprehensive summary of the available studies.

*Multiple non-critical weaknesses may reduce confidence in the review and it may be appropriate to lower the appraisal from moderate to low

**Table 4:** AMSTAR 2 items and results (*n* = 7 included systematic reviews)

AMSTAR 2 Item	Yes	Partial Yes	No
1. Did the research questions and inclusion criteria include the components of PICO?	7		
2. Did the report contain an explicit statement that review methods were established prior to conduct?	2	5	
3. Did the authors explain their selection of study designs for inclusion?			7
4. Did the authors use a comprehensive literature search strategy?	7		
5. Did the authors perform study selection in duplicate?	7		
6. Did the authors perform data extraction in duplicate?	5		2
7. Did the authors provide a list of excluded studies and justify the exclusions?		2	5
8. Did the authors describe the included studies in adequate detail?		6	1
9. Did the authors use a satisfactory technique for assessing risk of bias (RoB) in individual studies?		4	3
10. Did the authors report on the sources of funding for the studies included in the review?	6		1
11. If meta-analysis was performed, did the authors use appropriate statistical methods?	1		
12. If meta-analysis was performed, did the authors assess the potential impact of RoB on results?	1		
13. Did the authors account for RoB in individual studies when interpreting results?	3		4
14. Did the authors provide a satisfactory explanation for any heterogeneity observed?	2		5
15. If quantitative synthesis was performed, did the authors investigate publication bias?	1		
16. Did the authors report any potential sources of conflict of interest, including funding?	6		1

The most consistent critical flaws across reviews were: failure to explain the rationale for inclusion of specific study designs (Item 3; all seven reviews scored No); inadequate provision of a list of excluded studies with justification (Item 7; five reviews scored No); and insufficient discussion of heterogeneity (Item 14; five reviews scored No). Two reviews did not conduct any risk of bias assessment of their included primary studies [12] and [13], which represents a critical methodological flaw. These two critically low-rated reviews carry substantially reduced evidential weight and are reported here for completeness.

The older reviews [12, 13] have search cutoffs that predate more rigorous reporting standards. Their methodological limitations partly reflect the standards of the era, but the consequent limitations on evidential strength remain applicable.

### Methods of assessing tooth movement

Considerable heterogeneity existed across reviews in the methods used to measure OTM, precluding any direct quantitative comparison. The human-focused review (Zymperdikas et al. [11]) used clinical measurement in vertical and sagittal planes supported by panoramic radiographs, cone-beam computed tomography (CBCT), and periradicular films.

Animal study assessment methods included measurement on stone models with sliding callipers, lateral cephalometric radiographs, silicon impression plaster models magnified with a profile projector, digital scanned models with imaging software, and photographic measurement of relapse distances. Histological methods included haematoxylin and eosin staining, scanning electron microscopy, immunohistochemical analysis including RANKL, OPG, and cathepsin K staining [21], TRAP staining for osteoclast counts, and measurement of active bone-resorptive lacunae. Sarango-Quishpe et al. [17] used mesiodistal movement distance as the primary outcome measure for their meta-analysis. These animal methods are not directly translatable to clinical practice.

### Type of bisphosphonate and method of administration

#### Animal evidence

Six of the seven included reviews examined BP effects in animal models. A wide range of agents was investigated including risedronate, clodronate, zoledronate, alendronate, ibandronate, pamidronate, and the experimental compound 4-amino-1-hydroxybutylidene-1,1-bisphosphonate (AHBuBP) [22]. Routes of administration included systemic (subcutaneous, intraperitoneal, intravenous, and intramuscular) and local (subperiosteal injection, intrasulcular gel, and gelatin hydrogel systems). Sarango-Quishpe et al. [17] additionally included studies comparing BPs against osteoprotegerin (OPG) as a reference agent.

Across all six animal-focused reviews, BP administration was consistently associated with reduced OTM regardless of the specific agent or route. Among individual agents, risedronate showed the most consistent inhibitory effect, particularly with local subperiosteal or gelatin hydrogel delivery, producing dose-dependent reductions in both OTM and relapse [12, 16]. Clodronate produced concentration-dependent inhibition of OTM, with higher concentrations generating greater reduction [14, 23, 24]. Zoledronate applied locally in molar extraction sites significantly reduced subsequent tooth movement with associated bone preservation [13, 25, 26]. The meta-analysis by Sarango-Quishpe et al. [17] confirmed that BPs were more effective than OPG in reducing mesiodistal OTM in animal studies, and that OPG alone did not produce a statistically significant reduction in OTM.

The observation that risedronate shows the most consistent inhibitory effect is based on a descriptive synthesis of animal data rather than a formal head-to-head comparative analysis across reviews. This finding should be interpreted accordingly.

#### Human evidence

The sole human-focused review [11] assessed patients receiving systemic BPs: alendronate (*n* = 23), ibandronate (*n* = 3), and zoledronic acid (*n* = 2) via oral (*n* = 24) or intravenous (*n* = 2) routes. This review was rated moderate quality and included one retrospective cohort study and six case reports, the majority without control groups. Findings indicated slower tooth movement rates and compromised treatment outcomes, particularly in extraction cases where incomplete space closure and poor root parallelism were observed. Direct comparison with animal data is not appropriate given the fundamental differences in study populations, drug dosing, and clinical context.

### Sagittal and vertical tooth movement

#### Animal evidence

Multiple animal reviews reported reduced molar mesialisation following BP administration [12–14]. Zoledronate applied locally in molar extraction sites produced significant reduction in protraction movements with associated bone preservation [13].

#### Human evidence

Zymperdikas et al. [11] observed slower space closure rates in extraction cases, with residual spaces and inadequate root parallelism attributable to tooth tipping rather than bodily movement. In the vertical plane, anterior tooth intrusion and selective extrusion of residual roots appeared to be less adversely affected by BP use, based on isolated case reports. Attempts to correct a unilateral open bite in BP-treated patients resulted in uneven occlusal contacts. These findings derive from case reports and series without control groups and should be treated with caution.

### Orthodontic Anchorage

All six animal-focused reviews reported that BPs can enhance anchorage by reducing OTM at the site of local BP application [8, 12–16]. The effect appeared spatially targeted, with adjacent sites largely unaffected. In the comparative meta-analysis by Sarango-Quishpe et al. [17], BPs were significantly more effective than OPG in reducing mesiodistal movement, with OPG failing to achieve a statistically significant reduction on its own [27]. Among locally administered agents, clodronate administered at shorter intervals in lower doses produced good inhibitory effects [14, 23, 24]. Zoledronate combined with OPG near the first molar area effectively limited retraction of teeth supported by orthodontic mini-implants [13, 28, 29].

All evidence for BP-mediated anchorage enhancement comes from animal studies. No human study has evaluated this application [30]. Every included review concluded that further clinical trials are necessary before local BP administration can be recommended for anchorage purposes in human patients.

### Root resorption

#### Animal evidence

Findings on root resorption were contradictory across animal reviews. Makrygiannakis et al. [15] found a decrease in root resorption following alendronate administration but no significant difference with zoledronic acid compared to controls [31–33]. Earlier studies cited in Iglesias-Linares et al. [12] reported decreased root resorption with topical risedronate and systemic AHBuBP [34, 35]. Fernandez-Gonzalez et al. [13] described reduced root resorption with new bone formation particularly in the lower root third following local BP application [36, 37]. Conversely, some studies found no difference in root resorption and one described atypical hyperplastic cementum formation following systemic BP administration prior to appliance placement, which may reduce root protection [38]. Sarango-Quishpe et al. [17] noted that BPs may reduce root resorption tendency during OTM but cautioned that their inhibitory effect on acellular cementum formation could increase root surface vulnerability in some conditions. These discrepancies likely reflect genuine variation across BP types, dosages, and experimental protocols.

#### Human evidence

Zymperdikas et al. [11] found mild root resorption as a predominant radiographic finding in BP-treated orthodontic patients, alongside sclerotic alveolar bone changes and widened periodontal ligament spaces. These findings derived from case reports without control groups and without standardised resorption classification criteria, precluding definitive conclusions on severity or clinical significance.

The contradictory evidence on root resorption, combined with the predominantly animal study base and low-to-moderate quality of the available evidence, means that no clear clinical guidance on root resorption risk can be derived from the current literature.

## Discussion

### Principal findings and evidence hierarchy

This umbrella review of seven systematic reviews consistently identified that BP administration reduces OTM. This conclusion must be contextualised carefully: six of the seven included reviews were based exclusively on animal studies, and the evidence from human patients remains limited to a single systematic review comprising predominantly case reports without control groups [11]. The translational relevance of animal findings is uncertain, given differences in bone turnover rates, drug metabolism, and the nature of orthodontic force application between experimental animal models and human clinical practice.

### Animal evidence and its limitations

The consistent finding across all six animal-focused reviews that BPs reduce OTM regardless of agent type, route, or dose suggests a robust biological mechanism operating through osteoclast inhibition. The finding that local administration restricts movement at the application site without affecting adjacent teeth is potentially clinically relevant for anchorage control but requires human validation. This inhibitory effect was further quantified by Sarango-Quishpe et al. (2025) [17], whose meta-analysis of seven animal studies found BPs to be significantly more effective than OPG in reducing mesiodistal OTM [27], with OPG alone failing to achieve a statistically significant reduction.

A critical limitation of the animal evidence is the diversity of experimental protocols across studies, encompassing varying species, BP agents and dosages, routes of administration, methods of inducing OTM, and outcome measures. This heterogeneity, combined with the critically low AMSTAR 2 ratings of two of the animal reviews [12, 13] and the moderate ratings of the remaining four [39–41], means that individual study conclusions warrant cautious interpretation.

### Human evidence

The only human-focused review (Zymperdikas et al. 2020 [11], rated moderate quality) is the most clinically relevant evidence in this synthesis. Its findings of slower OTM, incomplete space closure in extraction cases, and radiographic changes including mild root resorption and sclerotic alveolar bone are consistent with the biological effects predicted from animal data. However, the included primary studies were predominantly case reports, which carry significant risks of reporting bias and selection bias, and lack control groups. No prospective human trial or randomised controlled trial was retrieved by any included review, primarily due to ethical constraints on withholding or prescribing medications for experimental purposes in orthodontic patients. The absence of controls in most human studies means that the magnitude and clinical significance of BP effects on OTM in human patients remain undefined.

### Anchorage and clinical implications

The potential use of locally administered BPs for anchorage reinforcement is an area of theoretical interest supported by consistent animal evidence. However, without human trial data, no recommendation for clinical use can be made. Clinicians managing orthodontic patients already receiving systemic BPs should be aware of the potential for compromised tooth movement, particularly in planned extraction cases, and should counsel patients accordingly. The known risk of medication-related osteonecrosis of the jaw (MRONJ) associated with BP use must also be considered in the clinical management of these patients, particularly where invasive dental procedures are planned [10].

### Methodological considerations

This umbrella review incorporated CCA assessment, AMSTAR 2 quality appraisal, and separation of animal and human evidence. Formal evidence grading frameworks such as GRADE were not applied, primarily because the heterogeneity of the evidence base would make such grading unreliable; the conclusions are accordingly more cautious than those of an overview in which formal grading is feasible. Authors undertaking future work in this area are encouraged to apply the PRIOR (Preferred Reporting Items for Overviews of Reviews) guidelines and to incorporate formal evidence grading when the available evidence base becomes more homogeneous [19, 20].

The decision to include both animal and human systematic reviews was deliberate and is justified by the extreme scarcity of human data. Excluding animal reviews entirely would have left a synthesis of a single review, providing insufficient context for interpretation. The key safeguard adopted was to present and interpret animal and human evidence separately throughout all sections, without integrating findings across these fundamentally different levels of evidence.

### Heterogeneity and synthesis limitations

The inability to perform a meta-analysis at the level of this umbrella review reflects genuine heterogeneity in primary study designs, species, BP agents, dosages, routes of administration, and outcome measures. Six of the seven included systematic reviews did not conduct a meta-analysis of their own primary studies; Sarango-Quishpe et al. [17] were able to do so only by restricting their analysis to a specific pairwise comparison between BPs and OPG in healthy animals, which limits the generalisability of that quantitative estimate. The structured synthesis employed here is an appropriate response to this challenge but carries lower evidential weight than quantitative synthesis. The degree of overlap identified in the CCA matrix (approximately 6.1%) indicates that several reviews, particularly those focused on local BP administration and anchorage, assessed overlapping bodies of primary evidence. This overlap does not invalidate the synthesis but means that independent confirmation of findings across reviews is partial rather than complete.

### Future research priorities

The most pressing research need is for well-designed prospective human studies or randomised controlled trials examining the effect of systemic BP therapy on orthodontic outcomes, with appropriate control groups and pre-specified outcome measures. Future studies should document BP type, dose, frequency, duration, and route of administration alongside patient characteristics including age, sex, and underlying bone metabolic diagnosis. Orthodontic variables including appliance type, initial malocclusion, and biomechanics should also be standardised or reported in sufficient detail to allow subgroup analysis. Standardised root resorption classification criteria would facilitate future synthesis across studies. Future systematic reviews and overviews in this field should adopt PRIOR guidelines and incorporate GRADE or equivalent evidence assessment [19, 20].

## Conclusions

Based on the available systematic review evidence, which derives predominantly from animal studies:

- BPs consistently reduce OTM in animal models regardless of the type of agent, dosage, or route of administration. This finding is supported by all six animal-focused reviews and was further supported quantitatively by a meta-analysis within one of those reviews, which found BPs to be significantly more effective than osteoprotegerin in reducing mesiodistal OTM. Risedronate showed the most consistent inhibitory effect, particularly with local administration, though this reflects descriptive rather than head-to-head evidence. These findings cannot be directly extrapolated to human clinical practice.
- The single systematic review of human patients reported slower tooth movement rates, incomplete space closure in extraction cases, and radiographic changes consistent with the known biological effects of BPs on bone, but these findings were based on predominantly case-level data without control groups.
- All evidence supporting the use of locally administered BPs for orthodontic anchorage reinforcement derives from animal studies and is insufficient to support clinical recommendations; evidence on root resorption is contradictory across both animal and human studies, and no definitive clinical conclusion can be drawn.
- Well-designed prospective human studies are needed to clarify the clinical implications of BP use during orthodontic treatment across all outcome domains.

## Supplementary Information

Supplementary Material 1.

## Data Availability

No datasets were generated or analysed during the current study.
