# Supplementary material for: Effect of bisphosphonates on orthodontic tooth movement: an umbrella review
Source: BMC Oral Health. 2026 Jul 2;26:1795. doi: 10.1186/s12903-026-08984-2 (PMC13599317; doi:10.1186/s12903-026-08984-2)
Supplement: Supplementary file 1 [file 12903_2026_8984_MOESM1_ESM.docx]

**Supplementary Table 1. Search strategy**

**PubMed**

| **No.** | **Search** | **Result** |
| --- | --- | --- |
| 1 | ("Bone resorption inhibitors" OR "Osteoclast inhibitors" OR "Antiresorptive agents" OR "Antiresorptive drugs" OR "Osteoporosis medications" OR "Bisphosphonate therapy" OR "Bisphosphonate treatment" OR "Bisphosphonate drugs" OR "Bisphosphonic acids" OR "Bisphosphonate compounds" OR "Bisphosphonate derivatives" OR "Bisphosphonate analogs" OR "Alendronate" OR "Risedronate" OR "Ibandronate" OR "Zoledronic acid" OR "Clodronate" OR "Pamidronate" OR "Etidronate" OR "Nitrogen-containing bisphosphonates") AND ("Orthodontic tooth movement" OR "Dental movement" OR "Tooth migration") | 41 |
| 2 | 1 AND "Systematic reviews" AND "Meta-analysis" | 5 |

**MEDLINE via OVID**

| **No.** | **Search** | **Result** |
| --- | --- | --- |
| 1 | Same combined strategy as PubMed | 41 |
| 2 | 1 AND "Systematic reviews" AND "Meta-analysis" | 5 |

**EMBASE**

| **No.** | **Search** | **Result** |
| --- | --- | --- |
| 1 | Combined bisphosphonate terms AND "Orthodontic tooth movement" | 49 |
| 2 | 1 AND "Systematic reviews" AND "Meta-analysis" | 1 |

**AMED (Allied and Complementary Medicine Database)**

| **No.** | **Search** | **Result** |
| --- | --- | --- |
| 1 | Combined bisphosphonate terms AND "Orthodontic tooth movement" | 5 |
| 2 | 1 AND "Systematic reviews" AND "Meta-analysis" | 0 |

**Cochrane Library**

| **No.** | **Search** | **Result** |
| --- | --- | --- |
| 1 | "Bisphosphonate" AND "Orthodontic tooth movement" | 5 |
| 2 | 1 AND "Systematic reviews" AND "Meta-analysis" | 0 |

**PsycINFO**

| **No.** | **Search** | **Result** |
| --- | --- | --- |
| 1 | Combined bisphosphonate terms AND "Orthodontic tooth movement" | 0 |
| 2 | 1 AND "Systematic reviews" AND "Meta-analysis" | 0 |

**Scopus**

| **No.** | **Search** | **Result** |
| --- | --- | --- |
| 1 | Combined bisphosphonate terms AND ("Orthodontic tooth movement" OR "Dental movement" OR "Tooth migration") | 78 |
| 2 | 1 AND "Systematic reviews" AND "Meta-analysis" | 20 |

**Web of Science**

| **No.** | **Search** | **Result** |
| --- | --- | --- |
| 1 | Combined bisphosphonate terms AND "Orthodontic tooth movement" | 59 |
| 2 | 1 AND "Systematic reviews" AND "Meta-analysis" | 11 |

**Supplementary Table 2. Study characteristics (full version) - Part 1**

| **Author; Year; Design** | **Country/Origin** | **No. studies** | **Sample size** | **Type of studies** | **RoB assessment tool** | **Databases searched** | **Period of search** | **AMSTAR 2 rating** |
| --- | --- | --- | --- | --- | --- | --- | --- | --- |
| Zymperdikas et al. 2020 SR | NR | 7 | NR | 1 retrospective cohort study; 6 case reports | ROBINS-I tool; two independent reviewers | MEDLINE, Scopus, ScienceDirect, Google Scholar, Web of Science, Cochrane, Ovid, VHL, Evidence-Based Medicine, Bandolier, African Journals Online, Library Genesis, ProQuest, ZB MED, metaRegister | Up to March 2019 | Moderate |
| Iglesias-Linares et al. 2010 SR | NR | 13 | 475 rats (one paper unclear) | 13 experimental animal studies | NR - no RoB assessment conducted | PubMed/Medline, Scopus, Ebsco Host, Cochrane | Up to December 2008 | Critically low |
| Fernandez-Gonzalez et al. 2015 SR | Korea (n=2), Japan, USA (n=4), Spain (n=2), Turkey (n=2) | 11 | 215 (36 mice, 179 rats) | 11 case-control animal studies | NR - no RoB assessment conducted | PubMed/Medline, Scopus, Cochrane | 2000 to July 2014 | Critically low |
| Harikrishnan and Ramasamy 2022 SR | NR | 7 | 310 animal samples (278 rats, 20 rabbits, 12 mice) | 7 case-control animal studies | SYRCLE's tool; majority uncertain RoB, one low RoB | PubMed/Medline, Scopus, Google Scholar, Cochrane | Up to May 2022 | Moderate |
| Makrygiannakis et al. 2019 SR | NR | 21 (2 BPs) | NR | 2 controlled animal studies assessing bisphosphonates | SYRCLE's Risk of Bias Tool | PubMed, Central, Cochrane, SCOPUS, Web of Science, Arab World Research Source, ClinicalTrials.gov, ProQuest | Until April 2018 | Moderate |
| Miranda et al. 2024 SR | Japan (n=1), China (n=1), Indonesia (n=1) | 3 | 171 rats, 75 guinea pigs | 2 experimental animal studies (split-mouth); 1 experimental-control study | SYRCLE protocol | PubMed, Web of Science, Ovid, LILACS, Scopus, Open Grey | April 2020; updated June 2023 | Moderate |
| Sarango-Quishpe et al. 2025 SR+MA | NR | 12 (SR); 7 (MA) | NR (healthy animals) | 12 animal studies (rats and mice) | SYRCLE tool; Egger's regression for publication bias | MEDLINE-PubMed, Scopus, Google Scholar, ScienceDirect, Taylor and Francis | Up to August 2023 | Moderate |

**Supplementary Table 2. Study characteristics (full version) - Part 2**

| **Author; Year** | **BP type and route of administration** | **Method of measuring tooth movement** | **Main findings** |
| --- | --- | --- | --- |
| Zymperdikas et al. 2020 | Alendronate (n=23), ibandronate (n=3), zoledronic acid (n=2). Oral (n=24) or intravenous (n=2). | Clinical measurement (sagittal and vertical planes). Radiographic: panoramic, CBCT, periradicular films. | Majority of cases showed slow progress of tooth movement and compromised results. Slower space closure in extraction cases and diastemata. Anterior tooth intrusion and selective root extrusion less affected. Radiographic findings: mild root resorption, sclerotic alveolar bone changes, widened PDL spaces. No significant difference in incisor alignment between BP users and controls. |
| Iglesias-Linares et al. 2010 | Risedronate (local injection, subperiosteal, subcutaneous); AHBuBP (subcutaneous); HEBP (subcutaneous); pamidronate (IV, n=2); clodronate (local); zoledronic acid (systemic infusion); alendronate (unknown route); cells with clodronate in vitro. | Measurements on plaster models with callipers; optical microscopy; histology (H&E staining); SEM; TEM; immunohistochemical analysis; TRAP histochemistry; DEXA for BMD/BMC; RT-PCR for COX-2 and RANKL; HPLC-Griess for NO; immunoassay for PGE2 and IL-1. | Consensus across included papers that OTM is reduced after BP administration. Risedronate most effective in reducing OTM, followed by AHBuBP then clodronate. Reduction in OTM relapse reported by several authors. In vitro study with human PDL cells found decreased mechanical stress, leading to reduced range of OTM. |
| Fernandez-Gonzalez et al. 2015 | Clodronate (local, n=2); zoledronate (local); bis-enoxacin (route NR); pamidronate (route NR); OPG (route NR). | NR in original review. | Dose-dependent reduction of molar movement observed. Zoledronate applied locally in extraction sites and mesial to second molars: significant reduction in tooth movement with bone preservation. Pamidronate: tooth movement inhibition with reduced osteoclasts on compression side. OPG identified as most potent inhibitor of osteoclastic function. |
| Harikrishnan and Ramasamy 2022 | Clodronate (subperiosteal, n=2); zoledronate (local, n=2); ibandronate (subperiosteal); risedronate (subperiosteal); one study route NR. | Scanned models with digital measurement software; lateral cephalograms; plaster model measurements; silicon impression models magnified x10 with profile projector. | All studies demonstrated decrease in OTM irrespective of BP type or dosage. Increasing clodronate concentrations inhibited OTM; shorter dosing intervals with lower concentrations produced better outcomes. Bisphosphonates limited OTM at the application site without affecting adjacent sites. More potent BPs in smaller doses or less potent BPs at higher frequency proposed to improve outcomes. |
| Makrygiannakis et al. 2019 | Alendronate [0.5 mg P/kg; subcutaneous; every other day]; zoledronic acid [0.1 mg/kg; intraperitoneal; once, 1 week before movement]. | Histomorphometry; H&E staining; measurement of resorption lacunae number and surface area (micrometres squared). | Decrease in osteoclastic activity following BP administration confirmed. Alendronate: decrease in root resorption vs controls. Zoledronic acid: no significant difference in root resorption vs controls. |
| Miranda et al. 2024 | Risedronate: subperiosteal injection adjacent to upper first molar; intraperitoneal injection; intramuscular injection (mesial subperiosteal); topical gelatin hydrogel application. | Stone models with sliding callipers (first molar intercrestal distance); histology with osteoclast counts; digital callipers; TRAP staining; immunohistochemistry (RANKL, OPG, cathepsin K); sliding callipers for relapse and interincisal distance. | Topical risedronate inhibited relapse movement in a dose-dependent manner. Risedronate slowed OTM in ovariectomised rats. Gelatin hydrogel with risedronate reduced relapse at seven days post-stabilisation in a dose-dependent manner. Hydrogel system delivered risedronate locally and in a controlled manner, showing potential for targeted orthodontic application. |
| Sarango-Quishpe et al. 2025 | Bisphosphonates (various types including nitrogen-containing and non-nitrogen-containing) vs osteoprotegerin (OPG). Administered during active OTM in healthy animals. | Mesiodistal OTM distance as primary outcome measure for meta-analysis. Risk of bias assessed using SYRCLE tool. Publication bias by Egger's regression. | BPs significantly more effective than OPG in reducing mesiodistal OTM in animal studies. OPG alone did not achieve a statistically significant reduction in OTM. SYRCLE RoB: mostly unclear risk across included studies. No significant publication bias detected by Egger's regression. Findings consistent with prior narrative reports confirming BP superiority for OTM inhibition. |

**Supplementary Table 3. Excluded studies and justification for exclusion**

| **Title and reference** | **Reason for exclusion** |
| --- | --- |
| Could medications and biologic factors affect post-orthodontic tooth movement changes? A systematic review of animal studies (Kaklamanos et al. 2021) | Did not meet inclusion criteria |
| Effect of bisphosphonates on orthodontic tooth movement - an update (Krishnan et al. 2015) | Not a systematic review |
| Non-invasive adjunctive modalities in accelerating orthodontic tooth movement: a review (Shahrin et al. 2021) | Not relevant |
| The potential use of pharmacological agents to modulate orthodontic tooth movement (Kouskoura et al. 2017) | Not a systematic review |
| The impact of bisphosphonates on orthodontic treatment (Poelmans and Carels 2012) | Not relevant |
| Osteoporosis, osteonecrosis, and orthodontics (Leung et al. 2009) | Not relevant |
| The role of bisphosphonates in orthodontic tooth movement - a review (Ajwa 2019) | Not a systematic review |
| Medication effects on the rate of orthodontic tooth movement: a systematic literature review (Bartzela et al. 2009) | Did not meet inclusion criteria |
| Effects of common medications on orthodontic tooth movement: a systematic literature review (Al Shayea 2021) | Did not meet inclusion criteria |
| Effect of bisphosphonates on orthodontic tooth movement in osteoporotic patients: a review (Arbelaez et al. 2018) | Not a systematic review |
| Effectiveness of biologic methods of inhibiting orthodontic tooth movement in animal studies (Cadenas-Perula et al. 2016) | Did not meet inclusion criteria |
| Effects of bisphosphonates on orthodontic treatment and the TMJ: a systematic review (Gupta et al. 2017) | Did not meet inclusion criteria |
| The effects of commonly used drugs on orthodontic tooth movement: a systematic review (Batmaraj and Umashankar 2014) | Did not meet inclusion criteria |
| Effects of bisphosphonates in orthodontic therapy: systematic review (Rodolfino et al. 2012) | Did not meet inclusion criteria |
| Bisphosphonates treatment and orthodontic considerations (Ghoneima et al. 2010) | Did not meet inclusion criteria |
| Orthodontics and bisphosphonates: risks, limitations, and considerations: a narrative review (Cunha et al. 2025) | Not a systematic review |
